# Quantitative traits of early-stage osteochondrosis lesions in porcine distal femurs are consistent with skeletal developmental age

**DOI:** 10.1093/jbmrpl/ziag091

**Published:** 2026-05-22

**Authors:** Brittney P Kokinos, Pamela J Lang, Laura A Amundson, Matthew A Halanski, Thomas D Crenshaw

**Affiliations:** Department of Animal and Dairy Sciences, University of Wisconsin-Madison, Madison, WI 53706, United States; Department of Orthopedics and Rehabilitation, Shriners Children’s Twin Cities, Woodbury, MN 55125, United States; Department of Animal and Dairy Sciences, University of Wisconsin-Madison, Madison, WI 53706, United States; Department of Child Health, University of Arizona College of Medicine-Phoenix, Phoenix Children’s Hospital United States, Pheonix, AZ 85016, United States; Department of Animal and Dairy Sciences, University of Wisconsin-Madison, Madison, WI 53706, United States

**Keywords:** endochondral ossification, ossification variants, primary and secondary center of ossification, developmental age, clinical imaging

## Abstract

Early-stage osteochondrosis (OC) lesions are focal failures of endochondral ossification in the physeal and epiphyseal regions. Lesions are initially detected and quantified as ossification variants using non-invasive imaging methods. The etiology of OC lesions is not understood, as early-stage lesions are rarely imaged in children or animals until symptoms advance to stages that require intervention. The distal femur, a common site for OC lesions, provides an easily accessible, anatomical location to study early-stage development. Non-invasive CT and MRI techniques for distinguishing early-stage lesions were compared in images collected from Cross-sectional and Longitudinal Studies. Osteochondrosis lesion traits in the physeal and epiphyseal regions of swine distal femurs were quantified. Cross-sectional subjects were randomly selected from asymptomatic, crossbred swine at 7, 12, and 24 wk of age. A longitudinal cohort was repeatedly imaged at 6, 12, and 18 wk of age. Subsets of femurs from each age group were selected for histological analysis. Ossification variants defined as abnormal physeal widths without defined OC lesions, were observed at 7 wk. At 12 wk, 2.7 ± 1.8 physeal lesions per pig with an average volume = 9.8 ± 10.0 mm^3^ were quantified but no epiphyseal lesions were detected. At 24 wk, similar numbers (2.1 ± 1.4) of epiphyseal and physeal lesions were observed. Epiphyseal lesions had greater volumes (44.5 ± 53.5 mm^3^). At 24 wk, atypical ossification variants not previously characterized, were unexpectedly observed in over 40% of the femurs. These large, undefined regions characterized by extreme undulations and irregular mineralization without physeal widening were labeled irregular physeal lesions. Although the quantitative outputs differed, both CT and MRI methods yielded consistent inferences regarding the developmental age and location (primary vs secondary centers of ossification) of OC lesion progression across Cross-sectional and Longitudinal Studies. These outcomes support future research efforts to identify pivotal molecular signals controlling the initiation, progression, and regression of early-stage lesion development.

## Graphical Abstract

**Graphical Abstract:**
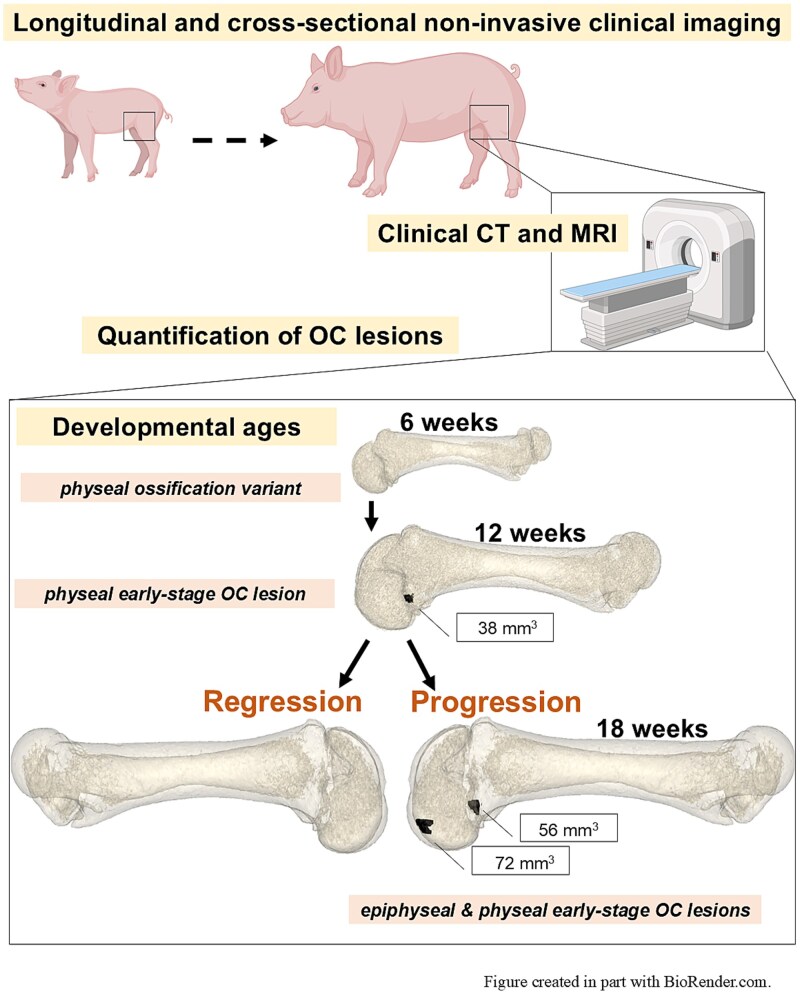

Clinical computed tomography (CT) and magnetic resonance imaging (MRI) techniques were applied in Longitudinal and Cross-sectional studies to scan femurs from pigs at three developmental ages. Images were analyzed to quantify the anatomical locations (physeal and epiphyseal regions) and the 3-dimensional size (volume and area) of osteochondrosis (OC) lesions. These techniques can be used to follow the developmental progression and regression of early-stage OC lesions.

## Introduction

Osteochondrosis (OC) lesions are focal failures in endochondral ossification (EO) that have similar clinical observations, histologic and radiologic features in humans,^1–3^ and other mammals.^4–6^ As multiple classification systems exist,^3^^,^^5^^,^^7^ herein advanced-stage lesions of OC without evident osteoarthritis are simply referred to as osteochondritis dissecans (OCD). Osteochondritis dissecans lesions are frequently characterized and clinically diagnosed, as OCD lesions are more symptomatic than early-stage OC lesions. Our current efforts are to understand the developmental age and regional locations of early-stage OC lesions in juvenile swine as a biomedical model.

In adolescents, epiphyseal OCD lesions are clinically detected after symptoms of joint pain, effusion, and varying degrees of physical dysfunction^8^^,^^9^ in the knee, elbow, or shoulder. In children the greatest incidence of lesions occurs in the knee, specifically in weight-bearing regions of the lateral posterior aspect of the medial femoral condyle (MFC) and, less frequently, the lateral femoral condyle (LFC) and patella.^9^ Untreated OCD lesions may progress to subchondral cysts, subchondral fractures, chondral fissuring, loose body formation, and osteoarthritis.^3^^,^^7^^,^^10^^,^^11^

Early-stage OC lesions may be detected and quantified using non-invasive clinical imaging methods, such as CT or MRI.^3^ Initial lesions, defined as ossification variants, are identified as regions of irregular cartilage widening without discrete boundaries, in physeal or epiphyseal regions. Ossification variants may progress to early-stage OC lesions detected as focal regions of non-mineralized cartilage matrix with discrete boundaries of retained cartilage in the subchondral bone along the physeal and epiphyseal regions. Few longitudinal studies have documented whether ossification variants or early-stage OC lesions pose a risk for progression to more severe OCD lesions.^7^^,^^9^ The molecular signals^2^^,^^10^ that initiate and allow the progression of early-stage lesions before clinical symptoms are not well documented.^9^^,^^11^ Assessments of only end-stage, OCD lesions preclude the detection of temporal molecular signals involved in the early stages of pathogenesis and advancement to more symptomatic lesions, or the detection of signals that mitigate lesion progression.

Animal models that consistently produce spontaneous, early-stage OC lesions are needed to unravel the developmental age and regional locations of early-stage OC lesion progression. Studies in swine have primarily focused on epiphyseal lesions^6^^,^^12^ with limited studies of physeal lesions. The integration of cellular signals and biomechanical properties between the physeal and epiphyseal regions was recently reviewed.^13^ Crosstalk between the 2 regions differentially affected tissue composition, biomechanical properties, and molecular signals during normal, age-related bone growth. The consequences of early-stage OC physeal lesions on the subsequent development of epiphyseal lesions are not established. Disruptions in the column formation of proliferative physeal chondrocytes, before the formation of a secondary center of ossification, altered the regional position of the secondary center of ossification and the subsequent modeling of long bones in mice.^14^ Physeal lesions are associated with the formation of physeal bars due to focal growth arrests that lead to abnormal long-bone growth and angular deformities in children.^15^^,^^16^ Angular deformities alter biomechanical load distribution and potentially the formation of epiphyseal OC lesions. Further evidence that physeal lesions may alter load-bearing regions with impacts on epiphyseal EO as a comorbidity in joint surface lesion development was supported by ex vivo measures of intra-articular pressures in swine.^17^ Peak pressures associated with joint extension exceeded thresholds for cartilage catabolism but not cell viability, supporting the biomechanical roles in OC lesion progression. Thus, non-invasive, clinical imaging methods in an animal model are needed to detect and follow the progression of early-stage OC lesions to delineate the interrelationships of regional lesions with developmental age. Initial efforts to image physeal lesions in juvenile swine were recently described using MRI techniques.^18^

Swine models are applicable in biomedical and nutritional studies, particularly for investigations of skeletal physiology, due to their skeletal size and molecular signaling similarities to humans.^19–21^ As the knee is the most common site for OC lesions in humans and similar lesions are described in swine distal femurs,^6^^,^^22^ the distal femurs of skeletally immature, crossbred swine were chosen to study the developmental patterns of early-stage OC lesions. In this study, 2 non-invasive methodologies were compared for the detection of early-stage lesions. Histological methods were used to illustrate the tissue traits of lesions detected by non-invasive methods. The current objectives were to: (1) determine the ages at which distinct early-stage OC lesions develop in the context of aged-related changes of whole femur development; (2) characterize regional locations (physeal vs epiphyseal) of these lesions; (3) compare distinctions in lesion traits assessed by clinical CT and MRI methods; and (4) provide histological characterizations of the observed lesions. These objectives will facilitate future examinations of the molecular signals involved in early-stage OC lesion development.

## Materials and methods

### Experimental design overview

Image analyses of clinical CT and MRI scans were used to quantify early-stage OC lesion traits in the distal femurs of skeletally immature pigs (6-24 wk of age), which correspond to adolescents at 4- to 12-yr of age.^21^ Separate experiments provided a Cross-sectional Study of different animals at 3 ages (7, 12, and 24 wk) or a Longitudinal Study of the same 6 animals at 3 ages (6, 12, and 18 wk). Subsets of femurs were collected for histological assessments from each age group.

### Animals

#### Cross-sectional Study samples

After the euthanasia of the juvenile pigs, their hind limbs were collected at designated ages for non-invasive imaging. The animal study was conducted at the University of Wisconsin-Madison Swine Research and Teaching Center (SRTC). All animal procedures were approved by the University of Wisconsin-Madison Institutional Animal Care and Use Committee (IACUC, protocol number A005856). Female and male pigs, equally balanced across age groups (*n* = 72 total) were produced by crossbred sows (Large White × Landrace) bred to Duroc boars. Males were castrated within 3 d of age per standard operational procedures for swine. After weaning (26.5 d of age, approximately 6 kg body weight), pigs were fed diets formulated to meet or exceed nutritional requirements^23^ for each growth phase from 20 to 120 kg body weight based on established SRTC standard diets. Pigs had free-choice access to feed and water throughout the study. Subsets of animals were randomly selected for euthanasia at 7 (*n* = 6), 12 (*n* = 20), or 24 (*n* = 46) wk of age. These ages were intentionally selected based on previous studies that reported a greater number of lesions at 12-14 wk than 24 wk of age.^24^^,^^25^ The current study included a younger age (7 wk) to further evaluate detection of early-stage lesions. After euthanasia, intact right femoral-tibial joints were collected, ensuring that surrounding tissues were retained to enhance image quality. Excised limbs were chosen rather than the entire animal to facilitate transportation from the animal facilities to the medical imaging facilities as an effort to reduce imaging costs.

#### Longitudinal Study samples

Six, 6-wk-old, crossbred female pigs from the SRTC herd were transferred to the campus Livestock Laboratory 1 wk before imaging procedures to allow for transport and housing adaptation. Throughout the trial, the pigs were fed SRTC standard control diets formulated to meet or exceed nutrient requirements for swine.^23^ Pigs were housed in an environmentally controlled facility throughout the trial period, except for brief (1-2 d) transfers at 6, 12, and 18 wk of age to the campus Wisconsin Institute for Medical Research (WIMR) facility for clinical CT scans. Due to transportation and logistics of animal movement, different ages (6 vs 7 wk and 18 vs 24 wk) of animals were imaged compared to that of the Cross-sectional Study. All animal procedures for the Longitudinal Study were approved by the IACUC (protocol number, A006268).

### Clinical imaging

Clinical CT and MRI scan protocols were developed with the technical assistance of the WIMR staff. Briefly, CT images were collected from scans at 120 kV with a slice thickness of 0.625 mm. MRI scans were performed using a 3 T MRI scanner (Single kV helical CT, GE Medical System, Waukesha, WI). Details of the CT and MRI instrument settings used to scan the excised limbs and live animals are reported in Tables S1 and S2, respectively.

#### Cross-sectional Study

Clinical CT scans were completed on all excised right hind limbs collected from pigs euthanized at 7 (*n* = 6), 12 (*n* = 20), and 24 (*n* = 46) wk of age, given short scan times and the ability to scan multiple hind limbs simultaneously. Five to 10 limbs per batch were placed in a medial-lateral position with the axial plane perpendicular to the head of the CT table. Images of single limbs were cropped and re-segmented for analysis, as subsequently described. Clinical MRI scans were completed on all hind limbs of pigs at 7 (*n* = 6) and 12 (*n* = 20) wk of age. However, at 24 wk, only 31 of the 46 limbs were scanned due to limited access to the MRI instrument. Limbs were individually placed in a knee coil oriented parallel to the head of the table.

#### Longitudinal Study

The same 6 pigs at 6, 12, and 18 wk of age were anesthetized using Telazol and Xylazine protocols. Clinical CT scans were restricted from the mid-spine to the mid-shaft of the tibia. Pigs were scanned in the sternal position with their hind limbs extended. Pigs were returned to the Livestock Laboratory until subsequent CT scans at 12 and 18 wk. Pigs were euthanized on the same day as the 18-wk scan. Due to the long scan times and limited access to the MRI instrument, no MRI scans were performed on live pigs in the Longitudinal Study.

### Clinical CT analysis

**Figure 1:**
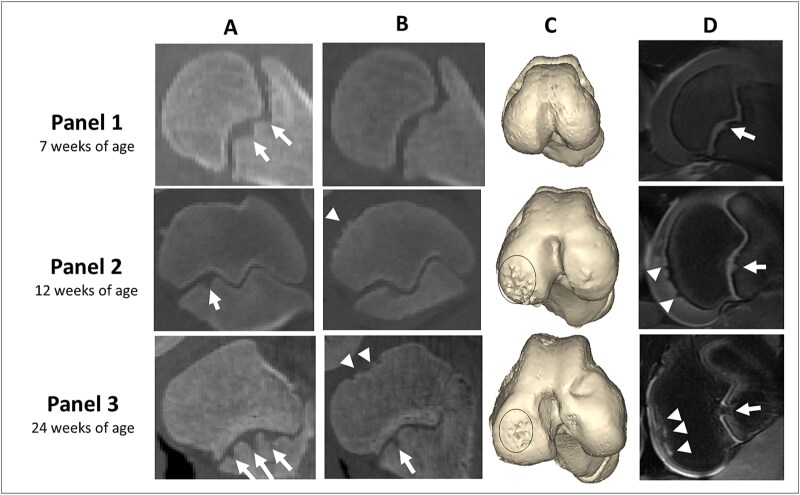
Cross-sectional study. Physeal and epiphyseal age-related changes in ossification. Swine distal femoral ossification variants and early-stage osteochondrosis (OC) lesions from CT scans of sagittal sections (columns A and B), and 3D reconstructed images (column C) and MRI scans of sagittal sections (column D). Ossification variants and OC lesions were identified in physeal (white arrows) and epiphyseal (white triangles) regions. Rows across each column are selected images from cross-sectional samples at 7, 12, and 24 wk of age (panels 1, 2, and 3, respectively). At 7-wk, CT images revealed ossifications variants, defined as irregular cartilage widening without discrete boundaries (panel 1, columns A and B). Likewise, MRI images exhibited some cartilage irregularities and bone signal changes along the physeal regions, (panel 1, column D). At 12 wk of age, (panel 2, columns A-D), show ossification variants as detected as irregular mineralization along the epiphyseal surface (white triangles). At 12 and 24 wk (panels 2 and 3), early-stage OC lesions, defined as discrete lesions detected as void regions in X-ray attenuation (columns A and B) for CT images or focal regions of retained cartilage in MRI images (column D) in the physis. At 24 wk, early-stage OC epiphyseal lesions are shown in panel 3, columns B and D. See Table 2 for a summary of quantitative lesion traits in the physeal and epiphyseal regions and Table 3 for lesion dimensions from CT and MRI analysis.

Raw Digital Imaging and Communications in Medicine files of images from clinical CT scans were reconstructed in Materialize Interactive Medical Image Control System (Mimics) software (Materialize Mimics NV, Version 23, Belgium) to quantify whole femur mineral density and early-stage OC lesions defined by discrete, void regions in X-ray attenuation in areas that should be mineralized. Lesions were cataloged into the physeal and epiphyseal regions across longitudinal sections (0.625 mm thick sections) of the distal femur as previously described by our research team.^25^^,^^26^ Additionally, ossification variants were defined as regions of irregular cartilage widening without discrete boundaries in the physeal and epiphyseal regions as illustrated in Figure 1. Quantitative analysis of lesion traits was completed by Brittney P. Kokinos. Briefly, CT image outputs in Hounsfield units (HU) were defined by the radiographic attenuation coefficient in each voxel. The voxel volume was 0.096 mm^3^ (0.391 × 0.391 × 0.625 mm). Masks for each bone were created to allow the selection of voxels within a range (200-1800 HU), previously defined for mineralized bone tissue of juvenile pigs.^25^^,^^26^ Defined bone density masks were applied to the images to quantify whole bone volume (cm^3^), bone density (g/cm^3^), physis volume (mm^3^), and physis area (mm^2^). The reconstruction from masks allowed for quantitative assessments of 3D lesion traits that included lesion number, volume (mm^3^), and surface area (mm^2^). Exact locations for each lesion were defined by *x*, *y*, and *z* coordinates from a defined anatomical location on the whole femur.

### Histology

After clinical scans, subsamples (*n* = 2/age group) were collected for the histological characterization of cellular traits to assess consistency with the early-stage OC lesions identified by non-invasive images. Distal femurs were cut into 3- to 5-mm thick sagittal slabs using an Isomet saw. Subsequently, each slab was fixed (approximately 48 h) in 10% neutral-buffered formalin, then decalcified by sequential changes of 15% EDTA solutions for 1-2 wk, as previously described.^27^ Decalcified slabs were submitted to the Translational Research Initiatives in Pathology Laboratories at WIMR for paraffin embedding, thin-sectioning (5 μm), and staining (H&E). Slides were imaged using the Aperio AT2 at 0.5 μm/pixel resolution at 40× magnification. Scanned image files were analyzed using QuPath software (v0.5.1). Observational traits of the histology specimens, as described in the figure legends, were included to illustrate lesion regions within the context of tissue and cellular morphology in the growth plate zones (resting, proliferative, hypertrophic, and chondro-ossification zones).

### Statistical analysis

Age-related responses of CT image traits (the number of lesions per bone, lesion volume, and lesion surface area) were initially assessed using regression analysis with mixed-model and general linear model procedures (SAS 9.4). Femurs from individual animals served as the experimental units. Traits from 7, 12, and 24 wk were considered fixed effects as independent events. A subset of 20 bones for MRI scans was selected to represent the range of outcomes previously observed in CT scans for both ages (12 and 24 wk) as no lesions were detected at 7 wk. For traits of continuous variables, plots of residual variances were used to verify that traits were normally distributed, thus meeting criteria for ANOVA analysis. Averages and SD were reported for all traits. Quantitative measures were expected to differ as outputs from the CT and MRI modalities are optimized to detect different tissues. Thus, observational comparisons among the imaging modalities were described in the context of reliance on these modalities as an aid in diagnostic discernment by clinicians. Statistical inferences to compare the 2 studies were not applied as the studies were conducted in different years, thus the animals were not selected from a contemporary group. However, the animals were derived from the same genetic lines maintained for generations within our research herd.

## Results

Results from the Cross-sectional and Longitudinal Studies provided observational inferences for distinctions among imaging methodologies and pig ages on early-stage OC lesion traits in the distal femur. Based on daily animal observations by trained animal care staff and periodic observations by researchers, no visual evidence of locomotion or lameness concerns was observed in any pig. Additionally, abnormal lesions (previously not observed by our lab) were identified in the physeal and metaphyseal regions of femurs from the Cross-sectional Study. As a unique category, we defined these abnormalities as irregular physeal lesions (IPL) and included further characterized from CT images and histological sections.

### Cross-sectional study

#### Whole-bone characteristics

As expected, the entire femur volume and area increased with age (6, 12, 24 wk of age), reflecting long-bone growth (Table 1). Femur volume is a measure of the 3-dimensional distribution of the mineral matrix that includes the marrow cavity. A linear increase (*p* < .001) in femur volume (8.95 cm^3^/wk) revealed a constant, linear modeling of the bone matrix between 7, 12, and 24 wk. However, bone area, which included only the amount of mineralized matrix independent of volume, did not accumulate at a constant rate across the ages sampled (quadratic response, *p* < .04). The initial rate of increase in bone area (14 cm^2^/wk) between wk 7 and 12, was less than the increase (37 cm^2^/wk) between wk 12 and 24. The initial lag in bone mineral area between 7 and 12 wk, as bone volume increased, described a distribution of bone mineral over a larger volume of bone, especially for the 12-wk age group. This distribution of mineralized tissue across a larger volume was consistent with an apparent reduction in bone density at 12 wk, although significant differences in bone density were not detected across the 3 age groups.

**Table 1:** Cross-sectional study. Entire femur traits from CT image analysis at 7, 12, and 24 wk of age^a^^-^^c^.

		**Age, wk** ^**d**^ ^**,**^ ^**e**^		
**Femur Traits**	**Units**	**7**	**12**	**24**
**Volume** ^**f**^ ^**,**^ ^**g**^	cm^3^	30.5 *±* 9.7	75.0 *±* 18.8	182.4 *±* 17.5
**Area** ^**f**^ ^**,**^ ^**h**^	cm^2^	211 *±* 48.3	280 *±* 57.4	725 *±* 123.2
**Density**	g/cm^3^	1.60 *±* 0.05	1.54 *±* 0.03	1.67 *±* 0.04

a Femurs collected from an equal number of female and male pigs within each age group at 7 (n = 6), 12 (n = 20), and 24 (n = 46) wk of age with traits from CT image analysis using Mimics software to determine bone volume (cm^3^), bone area (cm^2^), and bone density (grams of bone mineral/cm^3^).

b Whole-bone volume (cm^3^) was derived as the 3D spatial summation of voxels detected by Hounsfield units (HU) thresholds from 200 to 1800 HU. Whole-bone area (cm^2^) is the 2D summation of voxels.

c Bone density was calculated from the average HU using an equation derived from CT images of a fabricated phantom with known densities where CT bone density, g/cm^3^ = 0.00114 * HU + 1.06 as described by Collins et al.^26^

d Values are means *±* SD.

e Difference due to sex, *p* < .03. (Data not shown).

f Difference due to wk, *p* < .001.

g Regression with age where W = wk and Bone volume = −32.35 + 8.95*W, R^2^ = 0.92, linear response, *p* < .001.

h Regression with age where W = wk and Bone area = 228.8-12.1*W + 1.36*W^2^, R^2^ = 0.82, linear and quadratic responses, *p* < .04.

#### Age-related distal femur ossification variants and early-stage OC lesions

Clinical CT and MRI images collected at 7, 12, and 24 wk revealed similar age-related physeal and epiphyseal changes. Ossification variants, ie, regions of irregular physeal widening in the cartilage of the osteochondral interface near the epiphyseal surface, without discrete lesions were detected at younger ages prior to the detection of defined OC lesions (Figure 1). Characteristics of physeal and epiphyseal early-stage OC lesions, including the number, volume, and area of the lesions, were quantified from clinical CT images (Table 2).

**Table 2:** Cross-sectional study. Distal femoral physeal and epiphyseal early-stage osteochondrosis lesions detected by CT image analysis of excised specimens collected at 7, 12, and 24 wk of age.

			**Age, wk** ^**a**^ ^**,**^ ^**b**^		
**Traits**	Units	Region	7	12	24
**Femurs with lesions/total** ^**c**^	n	physeal	0/6	19/20	14/46
	n	epiphyseal	0/6	0/20	35/46
**Lesions per femur** ^**d**^ ^**,**^ ^**e**^	n	physeal	0	2.7 *±* 1.8	2.1 *±* 1.4
		epiphyseal	0	0	2.1 *±* 0.12
**Lesion volume**	mm^3^	physeal	-	9.8 *±* 10.0	9.8 *±* 15.5
		epiphyseal	-	-	44.5 *±* 53.5
**Lesion area**	mm^2^	physeal	-	35.9 *±* 29.8	32.6 *±* 38.1
		epiphyseal	-	-	133.4 *±* 136

a Values are means *±* SD.

b No differences were detected between traits at 12 and 24 wk.

c The number of femurs with detectable lesions/the total femurs observed in 6, 20, and 46 limbs of pigs scanned at 7, 12, and 24 wk of age, respectively using CT. See Figure 1 for illustrations of age-related physeal and epiphyseal changes in CT images. Figures 5 and 6 provide representative histological images of physeal and epiphyseal lesions, respectively.

d The total number of lesions per femur was used to calculate statistical inferences at each age, ie., only bones with lesions were included. Only quantitative values greater than 0 were used in the calculated traits for volume and area.

e No defined epiphyseal early-stage OC lesions were detected at 12 wk. However, ossification variants, presumed to be precursors of early-stage OC lesions, were evident. The regional distribution and dimensions of these lesions are reported in Table 3.

At 7 wk, physeal ossification variants were evident in the clinical CT scans (Figure 1; panel 1, column A). Likewise, the MRI scans (Figure 1; panel 1, column D) revealed regions of metaphyseal edema and focal regions of physeal widening. No discrete, early-stage physeal lesions were detected at 7 wk using either clinical CT or MRI scans. Conversely, at 12 and 24 wk, both clinical CT and MRI scans detected discrete early-stage OC physeal lesions (Figure 1; panels 2 and 3, columns A and D, respectively). Quantitative analysis of physeal early-stage OC lesions derived from clinical CT images revealed comparable lesion quantity, volume, and area at 12 and 24 wk (Table 2). Although lesion size did not change between 12 and 24 wk, the number of pigs with lesions in the selected groups was greater at 12 (*n* = 19) than at 24 (*n* = 14) wk. As a cross-sectional study design, inferences that the number of lesions regressed between 12 and 24 wk cannot be fully validated, as longitudinal scans were not made.

Distal femoral condyle epiphyseal age-related changes and ossification variants were observed by clinical CT and MRI (Figure 1; columns B and D, respectively). At 7 wk, no ossification variants or early-stage OC lesions were detected in epiphyseal regions using clinical CT images (Figure 1; panel 1, columns B and C). Similarly, no subchondral marrow or chondral signal changes were evident at 7 wk by MRI (Figure 1; panel 1, column D). At 12 wk, epiphyseal ossification variants and non-focal alterations in T2 marrow signal were detected with clinical CT and MRI, respectively, as illustrated in Figure 1; panel 2, columns B-D, with quantitative data in Table 3. MRI scans detected epiphyseal marrow signal changes in MFC of all 20, 12-wk specimens and 8 of the 20 LFC. At 24 wk, early-stage OC lesions along the epiphysis of the femoral condyles were detected as focal subchondral bone marrow edema and articular cartilage signal abnormalities by MRI (Figure 1; panel 3, column D. These lesions were associated with void regions in mineralization on clinical CT images (Table 3 and Figure 1; panel 3, columns A-C). Analysis of clinical MRI and CT images detected focal irregularities consistent with early-stage OC lesions in 18 of 20 specimens in the MFC and 9 of 20 specimens of the LFC (Table 3). Diameter dimensions were slightly larger for most MRI than CT lesions at 24 wk. For both MRI and CT images, lesion diameters were larger for the MFC than the LFC, consistent with the ossification variants observed at 12 wk (Table 3). Clinical CT and MRI modalities detected the same number (18 of 20) of femurs with epiphyseal lesions (Table S5). The regional distribution of lesions across the MFC and LFC was the same across imaging modalities. The epiphyseal lesions had more than 4× larger volumes than physeal lesions at 24 wk (Table 2). Additionally, subjective scores of the epiphyseal joint surface identified fewer lesions than were detected from CT scans (see Tables S3 and S4).

**Table 3:** Cross-sectional study. Comparisons of epiphyseal lesion measurements derived from MRI and CT image analysis of ossification variants and early-stage osteochondrosis lesions on the medial and lateral condyles of excised femurs collected at 12 and 24 wk of age ^a^^-^^c^.

				**Lesion dimensions by orientation, mm** ^**d**^		
**Femur regions**	**Age, wk**	**Mode**	**Count, *n***	**AP**	**ML**	**Depth**
**Medial Condyle**	12	MRI	20/20	14.1 *±* 2.7	11.1 *±* 1.9	2.4 *±* 0.7
	12	CT	18/20	12.7 *±* 3.8	11.1 *±* 3.4	2.8 *±* 0.7
**Lateral Condyle**	12	MRI	8/20	8.3 *±* 3.0	7.8 *±* 1.4	1.6 *±* 0.4
	12	CT	5/20	9.0 *±* 2.3	6.1 *±* 1.5	1.8 *±* 0.3
**Medial Condyle**	24	MRI	18/20	13.8 *±* 5.2	9.4 *±* 3.4	5.7 *±* 1.9
	24	CT	18/20	11.8 *±* 5.1	9.6 *±* 2.9	4.2 *±* 1.2
**Lateral Condyle**	24	MRI	9/20	10.7 *±* 4.2	8.1 *±* 1.1	5.2 *±* 1.5
	24	CT	9/20	9.2 *±* 2.8	7.4 *±* 1.3	3.3 *±* 1.2

a Traits included the number of regional ossification variants and early-stage OC lesions (Count) on the epiphyseal surface and measurements of lesion diameters from anterior to posterior (AP, coronal plane), medial to lateral (ML, sagittal plane), and depth (axial plane) of subchondral bone involvement. Lesion measurements are reported within regions of interest for the medial and lateral condyles. Measurements are compared between images from either MRI or CT scans.

b Lesions included ossification variants (12 wk), defined as irregularities in cartilage and subchondral bone on the epiphyseal surface, and early-stage OC lesions defined as discrete lesions detected as void regions in x-ray attenuation as focal regions of cartilage retention in subchondral bone. See Figure 1.

c A subset (n = 20) of the 46 femurs from CT scans of 24-wk femurs were selected based on the femurs submitted for MRI scans. The subset included comparisons of lesions from the same animals. See footnotes in Table S5.

d Measurement values are means *±* SD.

This difference between CT and MRI measurements is likely attributed to the larger slice thickness and lack of axial images that precluded full 3D reconstruction of MRI, which allowed false classifications as 2 small, distinct lesions rather than 1 continuous lesion in CT reconstructed images. With CT image analysis, 18 of 20 MFC and 5 of 20 LFC had detectable lesions. Lesion diameters were consistent with MRI lesion diameters (Table 3). Thus, clinical CT and MRI modalities allowed for a similar quantification of lesion dimensions (anterior to posterior, medial to lateral, and depth) in the 24-wk specimens. However, the MRI allowed for the assessments of lesions by detection of linear hyper-intense T2 signals, cystic changes, disruption of subchondral bone and chondral fissuring as described in Table S5.

#### Irregular physeal lesions

**Figure 2:**
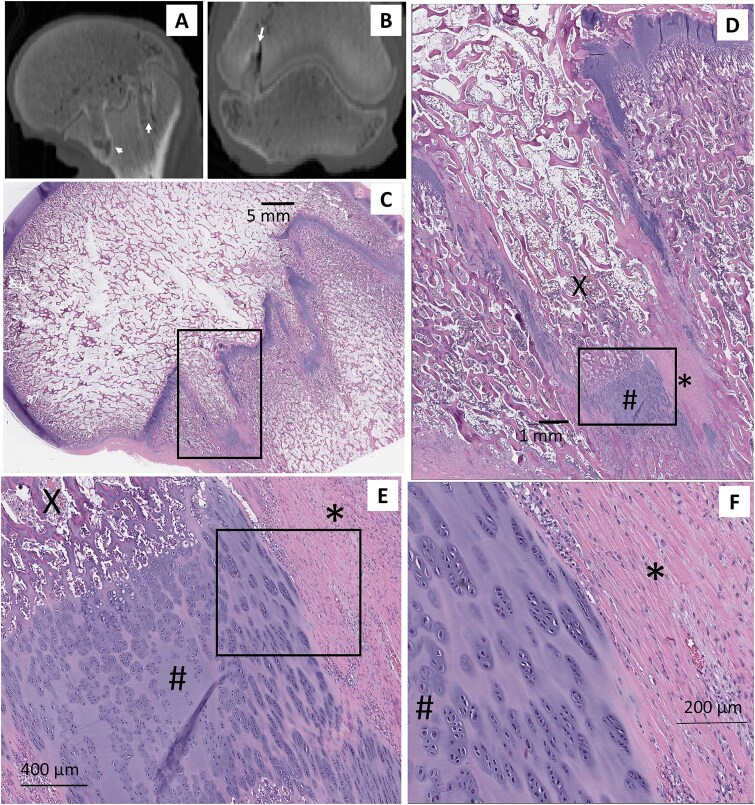
Cross-sectional study, irregular physeal lesions (IPL). IPL in the distal femur at 24 wk of age were identified in clinical CT images (panels A and B) by extreme undulations and irregularities in mineralization (white arrows) without physeal widening. Images A and B are sagittal and coronal views, respectively. H&E stained thin-sections (C-F) reveal regions of retained semi-disorganized hypertrophic chondrocytes (#), fibrotic tissue (*), and epiphyseal mineralized matrix (X) that extends into metaphyseal regions. See Table 4 for summary of IPL prevalence, regional distribution, and quantitative lesion traits.

In addition to the physeal and epiphyseal lesions detected by MRI and CT scans, unique lesions were observed at 24 wk in the Cross-sectional Study. These lesions were distinct from the previous early-stage OC physeal lesions, due to their irregular shapes with varied amounts of cartilage and mineralized matrix dispersed over a greater regional area than typical OC lesions that had defined boundaries in a focal region. Initially, these lesions were ignored. However, 20 of 46 excised distal femurs displayed these IPL. The IPL were distinguished by extreme undulations and irregularities in mineralization, without physeal widening (Figure 2; panels A and B). The IPL included irregular mineralized matrix regions dispersed in apparently unmineralized matrix regions (Figure 2; panels E-F). The IPL differed in their characteristics, as some regions had essentially no cartilage, with mostly an apparent mineralized matrix within the IPL area. Other IPL areas consisted of unmineralized cartilage. Consistent with OC lesions, more IPL were present in the MFC rather than in the LFC (Table 4).

**Table 4:** Cross-sectional study. Incidence and traits of abnormally large lesions referred to as irregular Physeal lesions (IPL), and associations with epiphyseal lesions in the distal femoral condyles and characteristics derived from clinical CT image analysis of specimens at 24 wk of age.

**Incidence of IPL** ^**a**^	**Units**	**IPL/Total, *n***	**Total, %**
**IPL / bone** ^**b**^	n	20/46	43.5
**IPL associated with epiphyseal lesion** ^**c**^	n	15/20	75.0
**Medial Femoral Condyle**	n	6/15	40.0
**Lateral Femoral Condyle**	n	4/15	26.7
**Both Condyles**	n	3/15	20.0
**Opposite Condyle**	n	2/15	13.3
***Lesion traits*** ^**d**^		**Mean**	**SD**
**IPL per femur**	n	0.9	1.1
**IPL volume**	mm^3^	113	177
**IPL area**	mm^2^	300	435

a See manuscript text for description of these unique irregular physeal lesions (IPL) and Figure 2 for histological images that illustrate the unique features of these lesions. IPL were only observed in femurs at 24 wk. These lesions were not included in the counts or traits reported for femoral physeal, early-stage OC lesions reported in Table 2.

b Values represent the number of lesions in the total number of femurs and percentage of distal femurs that had IPL at 24 wk (n = 46).

c Values represent the number and percentage of bones with IPL that were associated with epiphyseal, articular surface lesions within each respective condyle.

d Lesion traits of femurs with detectable IPL/the total femurs observed in 46 limbs of pigs scanned at 24 wk of age using CT. Only quantitative values greater than 0 were used in the calculated traits.

Compared with the physeal OC lesions in femurs from pigs at 24 wk (Table 2), the IPL regions had more than 11× larger volumes and 9× larger areas (Table 4). The timeline for the development of the IPL was not established in the current Cross-sectional Study. No IPL were observed in the 12-wk specimens, inferring that IPL developed between 12 and 24 wk, the age interval that bone area was dramatically expanding (Table 1). However, initial void regions in X-ray attenuation of physeal OC lesions that were present at 12 wk may have continued to progress and potentially converged to form IPL observed at 24 wk while in other animals, the smaller physeal OC lesions may have regressed to non-detectable lesions by 24 wk. These observations highlight the importance of longitudinal images collected from the same animals as described in subsequent sections.

### Longitudinal study

#### Age-related, whole bone, and physeal distal femoral traits

As observed in the Cross-sectional Study, bone volume and area increased with age, reflecting long bone growth (Table 5). In this cohort, the linear increase (*p* < .001) in femur volume (8.96 cm^3^/wk) was remarkably similar (8.95 cm^3^/wk) to the bone volume growth responses of femurs from pigs in the Cross-sectional Study. The femur area consistently increased by 36.8 cm^2^/wk across the 6- to 18-wk age groups. Likewise, bone density increased by 0.008 g/cm^2^ per wk across the age groups. Although tedious efforts were required, the physeal volumes and areas were measured in the femurs collected in the Longitudinal Study (Table 5). Interestingly, the femur physeal volume peaked at 12 wk and decreased by 18 wk. As derived from the regression analysis (see Table 5, footnote b), physeal volume was maximum at 14.1 wk and decreased over the remainder of the trial, consistent with the initial phases of physeal closure within the experimental time course. Assessment of the physeal area predicted a maximum area at 16.7 wk. Both measures of physeal traits were consistent with the initial stages of physeal closure within the experimental timeline even though the entire femur volume and area continued to increase.

**Table 5:** Longitudinal study. Femur whole-bone characteristics from CT image analysis at 6, 12, and 18 wk of age^a^^-^^d^.

		**Age, weeks** ^**e**^ ^**,**^ ^**f**^		
**Traits**	**Units**	**6**	**12**	**18**
**Femur Volume** ^**g**^	cm^3^	14.3 *±* 2.14	62.9 *±* 6.25	121.9 *±* 9.39
**Femur Area** ^**h**^	cm^2^	144 *±* 32	378 *±* 69	586 *±* 173
**Femur Density** ^**i**^	g/cm^3^	1.49 *±* 0.019	1.55 *±* 0.024	1.58 *±* 0.028
**Physeal Volume** ^**j**^	mm^3^	544 *±* 41	1157 *±* 188	1043 *±* 437
**Physeal Area** ^**k**^	mm^2^	1005 *±* 119	3335 *±* 354	3851 *±* 1440

^a^Values based on averages of 12 femurs (6 pigs with left and right femurs) collected from longitudinal, CT scans of the same pigs at 6, 12, and 18 wk of age. Traits from CT scans used Mimics software to determine bone volume (cm^3^), bone area (cm^2^), and bone density (grams of bone mineral/cm^3^).

^b^Whole-bone volume (cm^3^) was derived as the 3D spatial summation of voxels detected by HU thresholds from 200 to 1800 HU. Femur area (cm^2^) is the 2D summation of voxels.

^c^Bone density was calculated from the average Hounsfield units (HU) using an equation derived from CT images of a fabricated phantom with known densities where CT bone density, g/cm^3^ = 0.00114 * HU + 1.06 as described by Collins et al.^26^

^d^Physeal volume (mm^3^) and area (mm^2^) traits were derived from masks of the physeal region using Mimics software to include non-mineralized voxels in the physeal region.

^e^Values are means *±* SD. Difference due to wk, *p* < .001.

^f^Difference due to femur side (left vs. right), *p* > .10. (Data not shown).

^g^Regression with age, where W = wk and Bone volume = −41.2 + 8.96 * W, R^2^ = 0.98, linear response, *p* < .002.

^h^Regression with age, where W = wk and Bone area = −71.7 + 36.8 * W, R^2^ = 0.75, linear response, *p* < 0.001.

^i^Regression with age, where W = wk and Bone density = 1.44 + 0.008 * W, R^2^ = 0.73, linear response, *p* <0.001.

^j^Regression with age, where W = wk and Physeal volume = −795 + 284 * W−10.1 * W^2^, R^2^ = 0.52, linear &quadratic response, *p* < 0.01.

^k^Regression with age, where W = wk and Physeal area = −3138 + 842 * W −25.2 * W^2^, R^2^ = 0.70, linear &quadratic response, *p* < 0.01.

#### Detection of age-related distal femur physeal lesions

**Figure 3:**
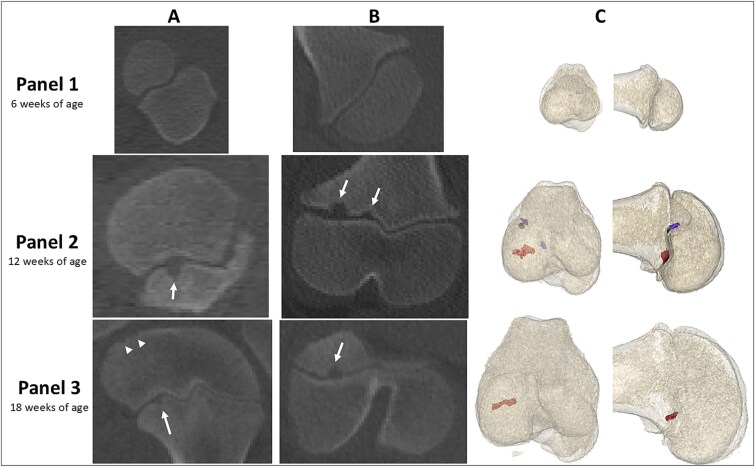
Longitudinal study. Longitudinal tracking of age-related changes in distal femoral physeal early-stage osteochondrosis (OC) lesions at 6, 12, and 18 weeks of age using clinical computed tomography (CT) scans of the same animal. Panel A and B are sagittal and coronal views, respectively. At 6 weeks, no discrete early-stage OC lesions were detected in physeal or epiphyseal regions (Panel 1, column A and B). At 12 and 18 weeks of age, distinct physeal early-stage OC lesions were identified as void regions in x-ray attenuation (Panels 2 and 3, column A and B) (arrows). No epiphyseal lesions were detected in any pig (n = 6) in the Longitudinal Study. Column C illustrates 3-dimensional reconstructed images of distal femurs from CT scans with distinct physeal lesions at 12 and 18 weeks. Unique color masks were used to facilitate tracing of the irregular shapes of lesions across the 0.625 mm sections to summate the lesion volume and area traits of individual lesions during reconstruction of the entire distal femur. See Table 6 for summary of quantitative lesion traits at each age.

Analogous to the cross-sectional observations, longitudinal clinical CT images delineated physeal age-related changes at 6, 12, and 18 wk (Table 6 and Figure 3; panels 1, 2, and 3, respectively). No early-stage OC lesions were detected using clinical CT images in the pigs at 6 wk of age. By 12 wk, clinical CT scans detected a greater average number (1.92, 1.50), volume (10.72, 6.64 mm^3^), and area (31, 23 mm^2^) of lesions compared to traits at 18 wk, respectively. As with the Cross-sectional Study, lesion number, volume, and area decreased from 12 to 18 wk. Physeal lesions were concentrated in the posterior regions of the MFC. Interestingly, no ossification variants nor distinct early-stage OC lesions were identified in the epiphyseal region of pigs in the Longitudinal Study, regardless of developmental age.

**Table 6:** Longitudinal study. Femoral physeal early-stage osteochondrosis lesion traits from CT images at 6, 12, and 18 wk of age^a^.

		**Age, wk**		
**Traits**	**Units**	**6**	**12**	**18**
**Femurs with lesions/total** ^**b**^ ^**,**^ ^**c**^	n	0/12	11/12	11/12
**Physeal lesions per femur** ^**d**^	n	0	1.92 *±* 1.14	1.50 *±* 0.67
**Lesion volume** ^**d**^	mm^3^	0	10.72 *±* 12.8	6.64 *±* 8.60
**Lesion area** ^**d**^	mm^2^	0	31 *±* 22.6	23 *±* 22.8
**Lesion width** ^**d**^	mm	0	3.69 *±* 1.63	3.49 *±* 2.88
**Lesion depth** ^**d**^	mm	0	2.16 *±* 1.01	1.87 *±* 0.91
**Sum of lesion volume** ^**d**^	mm^3^	0	27.1 *±* 24.1	12.6 *±* 10.1
**Sum of lesion area** ^**d**^	mm^2^	0	87 *±* 67.7	46 *±* 30.0

a Values based on averages of 12 femurs (6 pigs with left and right femurs) collected from longitudinal, clinical CT scans of the same pigs at 6, 12, and 18 wk of age. See Figure 3 for age-related changes from clinical CT images.

b The number of femurs with detectable physeal early-stage OC lesions / the total femurs observed at each age. Only quantitative values greater than 0 were used in the calculated traits.

c The total number of early-stage OC lesions per femur was used to calculate statistical inferences at each age, ie., only bones with lesions were included.

d Values are means *±* SD. Difference due to wk, *p* < .02.

One pig in the Longitudinal Study displayed a single IPL in the distal femur identified from the clinical CT images. No physeal ossification variants or widening were observed at 6 wk. However, at 12 wk of age, a focal area of cartilage retention was observed as a void region in X-ray attenuation (Figure 3, panel 2). By 18 wk, the lesion had become more irregular, with regions of mineralization paired with cartilage retention, like the IPL described in the Cross-sectional Study (Figure 2).

### Age-related histology and characterization of physeal lesions and IPL

**Figure 4:**
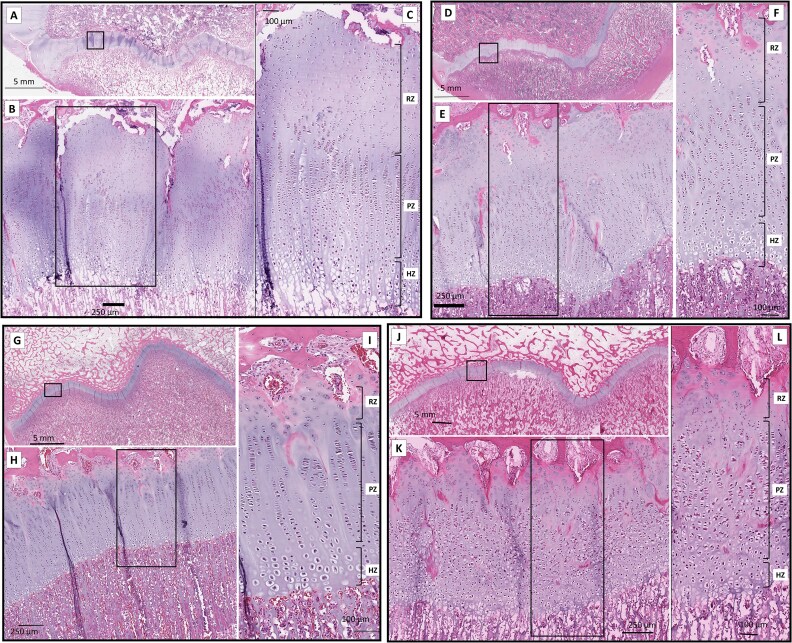
Normal age-related changes in the physeal region illustrated with H&E-stained thin sections. Distal femoral physis at 6-wk (A-C), 12-wk (D-F), 18-wk (G-I), and 24-wk (J-L) of age. Enlarged sub-panels within each age group show matrix and cellular morphology across physeal zones. These zones include: (1) the resting zone (RZ), which include small, round chondrocytes sparsely distributed within a dense extracellular matrix, that serves as a reservoir of progenitor cells; (2) the proliferative zone (PZ), where chondrocytes undergo rapid mitotic division and align into well-organized longitudinal columns, separated by flattened lacunae; (3) the hypertrophic zone (HZ), composed of large, chondrocytes embedded in a cartilage matrix; and (4, not labeled) the zone of primary calcification, where the cartilage matrix initially becomes mineralized, forming a scaffold for bone mineral deposition. The zonal organization reflects active longitudinal bone growth through regulated chondrocyte proliferation, maturation, and ossification. Focal failure in endochondral ossification in the physeal region results in early-stage osteochondrosis (OC) lesions as illustrated in Figure 5.

**Figure 5:**
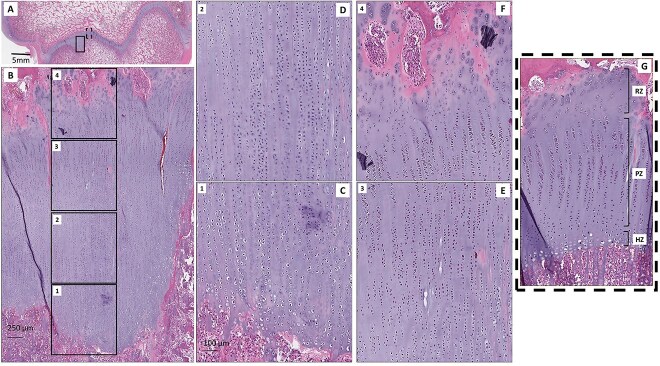
Comparisons of physeal zones in lesion and non-lesion focal regions. Histological and cellular morphology (Hematoxylin and Eosin-stained thin (5 µm) section) of the distal femur. Panel A illustrates an early-stage OC lesion (black box) that was detected from CT images (Figure 3) that included a focal failure in endochondral ossification in the physeal region in an 18-week-old distal femur. The lesion region (solid-line black box, enlarged in Panel B) compared to an adjacent non-lesion physeal region (Panel G, dashed-line yellow box) shows physeal zones as defined in Figure 4. Further enlargement of these sub-zones (Panels C-F) show chondrocyte cellular alignment and morphology. The lesion area is observed to be primarily retained proliferative and hypertrophic chondrocytes (Panels D-E). At the physeal-metaphysis interface (Panel C.1.) the columnar hypertrophic chondrocyte alignment is slightly lost compared to control HZ arrangement (Panel G, HZ).

Representative sagittal H&E-stained, decalcified sections of the distal femoral physis revealed the classic zonal architecture of EO at 6, 12, 18, and 24 wk of age. From the epiphyseal surface (top of image) to the metaphyseal region, the physeal region exhibited distinct zonal matrix and cellular morphology (Figure 4, panels C, F, I, and L). These characteristics are defined as the: resting zone (RZ); proliferative zone (PZ); hypertrophic zone (HZ); and the zone of primary calcification. Images from pigs at 6, 12, and 18 wk revealed an overall thinning of the physis, associated with age as previously described^28^ (Figure 4; panels A-I). This physeal thinning is consistent with the quantitative measures of physeal area and volume traits from CT images (Table 5). The 24-wk sample revealed disorganization of the PZ and a transition phase (pre-hypertrophic chondrocytes) to HZ, suggesting potential pre-closure of the physis (Figure 4; panels J-L). In physeal lesion regions retention of both the PZ and the pre-hypertrophic chondrocytes were evident (Figure 5, black boxes; panels A-F), compared to adjacent, non-lesion regions (Figure 5, yellow box; panels A and G) in a representative sample from femurs at 18 wk.

**Figure 6:**
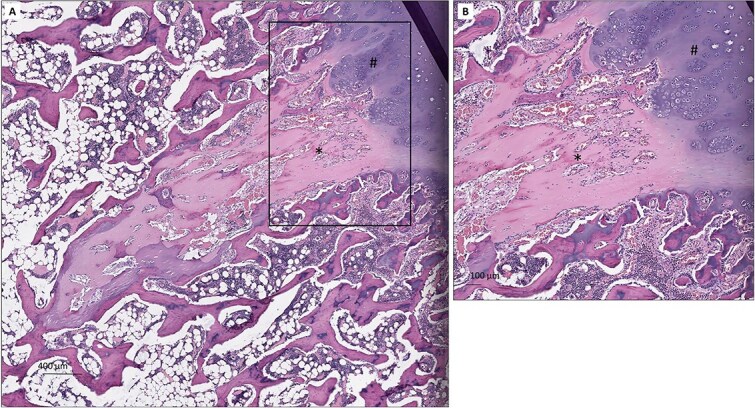
Thin-sections of an epiphyseal lesion in a 24-wk distal femur (H&E-stained section). Images show histological and cellular morphology of a focal failure in endochondral ossification in the epiphyseal region in a 24-week-old distal femur. Regions of retained necrotic and fibrosis cartilage (Panel A, lighter pink shades (*) extend from beneath articular cartilage on the joint surface into the epiphyseal subchondral bone. The lighter shaded regions (*) (Panel B) have a reduced number of chondrocytes dispersed in the cartilage matrix, compared to the above retained hypertrophic cells (#), consistent with lesions of osteochondrosis.

Epiphyseal early-stage OC lesions at 24 wk (Figure 6) in our swine model were histologically consistent with previous descriptions.^13^^,^^29^^,^^30^ The epiphyseal lesions exhibited necrotic, fibrotic regions that span deep into the subchondral bone with only limited amounts of retained hypertrophic chondrocytes. The cellular morphology of the epiphyseal lesions (Figure 6) differed from that of the physeal OC lesions (Figure 5) and differed from that of the IPL (Figure 2). The IPL revealed areas of retained physis (primarily retained PZ and HZ) intermingled with fibrotic collagenous tissue that mainly encompassed regions of apparently retained epiphyseal bone within the metaphysis.

## Discussion

The age-dependent expression of lesions associated with regional locations demonstrates potential opportunities and limitations of CT and MRI methodologies for the identification of early-stage OC lesions in a juvenile swine model. The initial detection of ossification variants followed by physeal and epiphyseal early-stage OC lesions that developed prior to the onset of obvious clinical symptoms were identified by both CT and MRI methodologies. Both the cross-sectional and longitudinal cohorts showed similar developmental age-related patterns of lesion traits in the distal femur. As the pathophysiology, clinical presentation, radiographic changes, and histological appearance of early-stage OC lesions are similar in humans and animals, these data are consistent with the use of juvenile swine as a model to study the early-stage development of OC lesions. These observations are consistent with earlier studies that incorporated MRI methods to assess epiphyseal and physeal OC lesions.^12^^,^^13^^,^^29^ The current results extend non-invasive methodologies to allow for quantitative assessments of lesion location, volume, and area traits using CT or MRI. As such, quantitative traits can be applied to track the progression or regression of lesions from early- to advanced-stages of development and to track the efficacy of treatment interventions.

In our swine study, clinical CT allowed for shorter scan times for image collections than MRI. With acknowledgement that CT scans impose increased radiation exposure in clinical applications with children,^3^ the shorter CT vs MRI scan times in swine allow for reductions in research expenses with equal or better quantitative results. However, significant fundamental differences exist between the clinical CT and MRI modalities.^31^ These differences must be considered, as early-stage OC lesions are located at the cartilage-bone interface. Clinical CT images provide better resolution for mineralized tissue, as the calcium concentrations in bone attenuate X-rays. In contrast, cartilage is better visualized using MRI due to the greater water (hydrogen proton) content.

Results from these Cross-sectional and Longitudinal Studies provided quantitative insights into the location (primary vs secondary centers of ossification) of age-related changes in ossification variants to follow the progression of early-stage OC lesions in swine distal femurs. Physeal-widening ossification variants were observed at 7 wk in the distal femurs, but no detectable, defined early-stage OC lesions were present. The greatest number and largest volume of defined OC physeal lesions were detected in the distal femurs at 12 wk. In contrast, fewer physeal lesions with smaller volumes were observed at later ages (18-24 wk). These observations are consistent with previous studies that concluded physeal lesions regressed with age.^22^^,^^32^ However, only limited data are available in swine to determine if physeal lesions cause abnormal long-bone growth and angular deformities^15–17^ with comorbidities associated with the formation of epiphyseal lesions.

Many studies of OC lesions have focused on epiphyseal lesions^3^^,^^4^ as these are linked to pain and lameness symptoms that appear at older ages, after the physis has narrowed and started to fuse. In the Cross-sectional Study, abnormal epiphyseal ossification variants were initially observed in regions of subchondral bone in the distal femurs at 12 wk, along with an increased number and volume of defined epiphyseal early-stage OC lesions at 24 wk. However, in the longitudinal trial, no epiphyseal ossification variants nor defined OC lesions were observed at 12 or 18 wk. These age-dependent, developmental patterns support that physeal lesions develop between 7 and 12 wk and epiphyseal lesions develop between 12 and 18 to 24 wk. Interestingly, within these ages none of the pigs with lesions displayed lameness symptoms or observable gait changes. An ongoing debate involves the challenge to determine which lesions are symptomatic, as correlations between OC lesions and conformation or locomotion traits^33^ and pain^34^ have not been established. Furthermore, the molecular signals that allow some ossification variants to progress into lesions while others regress with age are not defined.^8–11^ Consequently, future research must develop techniques that include more frequent non-invasive imaging, coupled with the analysis of molecular signals to track the early-stage development and subsequent progression of physeal and epiphyseal lesions.

The IPL detected in physeal regions at 24 wk has not, to our knowledge, been previously described in swine. The high incidence (>40% of femurs examined) of IPL, along with overall subchondral bone abnormalities, that included extreme irregular undulations without physeal widening, warrants further characterization. These IPL have similarities to physeal bars in children^15^^,^^16^ and may represent a variant of OC lesion progression. A greater number of IPL with larger volumes were observed in the current experiments compared to early-stage OC physeal lesions previously characterized by our laboratory.^25^ The tissue composition of physeal early-stage OC lesions and IPL observed in the swine femurs are apparently different (Figures 2 and 5, respectively). Early-stage OC lesions are primarily retained PZ and HZ cartilage zones, whereas IPL include both retained hypertrophic cartilage and increased fibrotic and reduced cellular regions that have apparently inhibited the replacement of epiphyseal with metaphyseal bone during EO, with a resultant irregular mineralization pattern. Future studies should include additional histological, immunohistochemical, and molecular characteristics of the IPL.

Overall, the current results revealed that the number and volume of physeal early-stage OC lesions decreased with age. Conversely, epiphyseal early-stage OC lesions increased with age, indicating an age-by-location interaction. These results are consistent with long bone growth in fetal and neonatal developmental stages,^35^^,^^36^ as the primary center of ossification (physis) is developmentally older than the secondary center of ossification (epiphysis). Thus, developmental age is a critical variable to consider for inferences of OC lesion development. Further support for differences due to developmental age is implied by results from recent efforts^18^^,^^22^ to track physeal lesions in young pigs. Physeal lesions were detected by MRI as early as 4 wk in the humerus,^18^ while in our studies with femurs, a developmentally younger bone, lesions were not detected until after 7 wk of age. Attempts to track early-stage OC lesion development in other bones such as ribs or vertebrae, have not been reported, although we have tracked longitudinal retention of kyphosis, associated with OC lesions in our hypovitaminosis D swine model.^27^ Our current results and future interests focus on the early-stage development of lesions. Ossification variants in the physis warrant research to identify the molecular signals associated with lesion initiation. Applications of non-invasive image modalities (clinical CT and MRI) will facilitate advancements in understanding the developmental patterns of lesion formation, progression, or regression. Although clinical imaging modalities are invaluable for non-invasive assessment, these methodologies lack the resolution necessary to detect early-stage lesions at the cellular and molecular levels. Although an invasive procedure, histological analysis coupled with immuno-histological and molecular methods provides a visualization of tissue architecture and cellular integrity, which allow for the identification of subtle changes that precede radiographic detection. This enhanced sensitivity supports histological and molecular procedures as critical tools for characterizing early-stage focal failures in EO.

In previous studies, OC lesions observed in physeal and epiphyseal regions were histologically different.^4^^,^^30^ However, the initial molecular signals of physeal vs. epiphyseal focal failures in EO must be further studied. Focal failures of EO in the epiphysis were histologically described as a consequence of cartilage necrosis and fibrosis, whereas focal failures in the physeal region were associated with altered development of the pre-hypertrophic and turnover of hypertrophic chondrocytes.^4^^,^^13^^,^^37^ The current study was not designed to definitively verify these regional histological distinctions, although our limited observations are consistent with previously reported data. Recent CT and MRI methodologies^31^^,^^38–40^ coupled with the future identification of molecular signals offer approaches to clarify the dynamic processes involved in EO.

This is the first study conducted using our swine model to histologically characterize lesions identified by clinical CT and MRI methods to assess the physeal and epiphyseal lesions in the distal femur. The “age” or stage of the lesion apparently influences its histological features, as older lesions contain more fibrosis, reduced cellularity, and increased necrotic tissue than early-stage lesions. Therefore, further histological characterization of these early-stage OC lesions is essential to gain deeper insights into the molecular signals involved in the initiation and continued progression.

Limitations of both the Cross-sectional and Longitudinal Studies must be acknowledged. Primary limitations include the number of observations and the infrequent intervals of the CT scans. The cross-sectional study design constrained the interpretation of ossification variants in the physeal and epiphyseal regions at 7, 12, and 24 wk of age. For example, the initial physeal ossification variants observed at 7 wk cannot be assumed to be precursors of the early-stage OC physeal lesions noted at 12 wk. Evidence to validate that ossification variants observed in younger animals may have resolved with age and bone growth, or if the early-stage lesions detected progress to more pathological lesions^9–11^ cannot be documented from the current studies. The imaging results revealed more epiphyseal lesions in older animals. However, the interval between 12 and 24 wk is too extensive to pinpoint the ages at which these lesions originated. Thus, cross-sectional studies restrict the investigation of lesion progression. The Longitudinal Study corroborated age-related changes in bone modeling, as characterized by whole bone density, volume and area traits, the locations of OC lesions (primary vs. secondary centers of ossification), and the regression of lesions as inferred in the Cross-sectional Study. Direct relationships between bone density and lesion progression could not be determined in the current results, although CT methods support the potential for studies of these relationships. Therefore, future longitudinal studies should involve a greater number of observations with more frequent intervals of CT scans.

## Conclusion

This study demonstrates that early-stage, pre-symptomatic OC lesions were detected in the swine distal femurs with clinical non-invasive imaging methods. These results advanced our understanding of the age-related, developmental changes of OC lesions in swine and provided distinctions between non-invasive image methodologies. Ossification variants were detected at younger ages in the primary center of ossification before OC lesions appeared in the secondary center of ossification. Furthermore, some lesions progressed with age while others regressed. Understanding the molecular signals in the progression and regression of lesions will require further studies. Our future efforts will use the time course and location of lesions established in this study to evaluate molecular signals and protein distributions within regions of focal failure in EO by comparing lesion and non-lesion regions of interest.

## Supplementary Material

Table_S1_ziag091

Table_S2_ziag091

Table_S3_ziag091

Table_S4_ziag091

Table_S5_ziag091

Final_update_R2_5_15_2026_Supplemental_Tables_ziag091

## Data Availability

All reasonable requests for review of datasets can be made through contacting the corresponding author following publication.
