# Supplementary material for: Quantitative traits of early-stage osteochondrosis lesions in porcine distal femurs are consistent with skeletal developmental age
Source: JBMR Plus. 2026 May 22;10(7):ziag091. doi: 10.1093/jbmrpl/ziag091 (PMC13318846; doi:10.1093/jbmrpl/ziag091)
Supplement: Table_S1_ziag091 [file table_s1_ziag091.docx]

Table S1. Clinical computed tomography (CT) imaging protocol for porcine limbs and live scans^1^.

| Single kV helical CT, GE Medical System, Waukesha, WI | |
| --- | --- |
| Scan Type | Helical |
| Detector Coverage (mm) | 40 |
| Beam Collimation (mm) |  |
| Detector Rows | 64 |
| Detector Configuration | 64 x 0.625 |
| Scan FOV | Large Body |
| Rotation Time (sec) | 0.5 |
| Helical pitch | 0.516 |
| kV | 120 |
| Smart mA or Manual mA | Smart mA |
| Noise Index | 12 |
| Slice Thickness (mm) | 5 |
| Interval (mm) | 2.5 |
| Recon kernel | Standard |
| DFOV | Please adjust according to the object size |
|  |  |
| Second Recon (Helical CT Thin) |  |
| Slice Thickness (mm) | 0.625 |
| Interval (mm) | 0.625 |
| Recon kernel | Bone plus |
| W/L | 3000/300 |

1. Computed tomography (CT) imaging protocol settings were utilized to scan excised limbs collected from porcine specimens at 7, 12, and 24 weeks of age.
