# Supplementary material for: Quantitative traits of early-stage osteochondrosis lesions in porcine distal femurs are consistent with skeletal developmental age
Source: JBMR Plus. 2026 May 22;10(7):ziag091. doi: 10.1093/jbmrpl/ziag091 (PMC13318846; doi:10.1093/jbmrpl/ziag091)
Supplement: Table_S2_ziag091 [file table_s2_ziag091.docx]

Table S2. Magnetic resonance imaging (MRI) protocol for porcine limbs^1^.

Set up = 3 Tesla, 32 channel Torso Coil FULL- lined with chux; Pig prone, feet first

To view in PACS (Radiology Solutions Radiology Station, Change Healthcare, 2019)

***Sequence-Specific Instructions:***

3-plane Localizer – Verify you have the correct side selected (ie, right vs left - start with knee of interest) Offset approximately R or L 110, 48FOV

Coronal PD - Center over knee joint on all 3 planes, cover all bony anatomy, 16FOV, make sure to include OC lesion

Coronal FS Intermediate-Weighted

Sagittal PD - Center over knee joint on all 3 planes, cover all bony anatomy, 16FOV, make sure to include OC lesion

Sagittal FS T2

1. Magnetic resonance imaging (MRI) protocol settings used to scan excised limbs collected from porcine specimens at 7, 12, and 24 weeks of age.
