# Supplementary material for: Quantitative traits of early-stage osteochondrosis lesions in porcine distal femurs are consistent with skeletal developmental age
Source: JBMR Plus. 2026 May 22;10(7):ziag091. doi: 10.1093/jbmrpl/ziag091 (PMC13318846; doi:10.1093/jbmrpl/ziag091)
Supplement: Table_S3_ziag091 [file table_s3_ziag091.docx]

Table S3. Osteochondrosis grading scheme for gross articular surface lesions in growing pigs^1^.

| Score | Description |
| --- | --- |
| 0 | Normal |
| 1 | Surface irregularities but no definitive lesion |
| 2 | Articular cartilage collapse/indentation < 5 mm diameter/length |
| 3 | Articular cartilage collapse/indentation > 5 mm and < 10 mm diameter/length |
| 4 | Articular cartilage collapse/indentation > 10 mm diameter/length |
| 5 | Articular cartilage clefting |
| 6 | Articular cartilage clefting with displaced fragment |

1. From Toth et al., *J Anim Sci*. 2016;94(9):3817-3825. doi:10.2527/jas.2015-9950.
