# Supplementary material for: Quantitative traits of early-stage osteochondrosis lesions in porcine distal femurs are consistent with skeletal developmental age
Source: JBMR Plus. 2026 May 22;10(7):ziag091. doi: 10.1093/jbmrpl/ziag091 (PMC13318846; doi:10.1093/jbmrpl/ziag091)
Supplement: Table_S4_ziag091 [file table_s4_ziag091.docx]

Table S4. Cross-sectional Study. Distribution of subjective visual lesion scores of articular surface lesions in the distal femur at 24 weeks of age^1^.

| Lesion score ^2^ | MFC ^3^, n | LFC ^3^, n |
| --- | --- | --- |
| 0 | 2/20 | 8/20 |
| 1 | 2/20 | 1/20 |
| 2 | 4/20 | 4/20 |
| 3 | 6/20 | 5/20 |
| 4 | 5/20 | 2/20 |
| 5 | 1/20 | 0/20 |

1. Values represent the number of femurs within each respective condyle location assigned to the respective lesions score of 20 femurs.

2. Subjective scores were assigned a value from 0 to 5 as described in Table S3.

3. MFC, medial femoral condyle; LFC, lateral femoral condyle.
