# Supplementary material for: Quantitative traits of early-stage osteochondrosis lesions in porcine distal femurs are consistent with skeletal developmental age
Source: JBMR Plus. 2026 May 22;10(7):ziag091. doi: 10.1093/jbmrpl/ziag091 (PMC13318846; doi:10.1093/jbmrpl/ziag091)
Supplement: Table_S5_ziag091 [file table_s5_ziag091.docx]

Table S5. Cross-sectional Study. Additional characterization derived from MRI analysis of epiphyseal early-stage lesions on either the medial or lateral condyle of excised femurs collected at 24 weeks of age^1^.

| MRI Traits | Observations ^1,2^, n | % ^3^ |
| --- | --- | --- |
| Lesions detected by MRI | 18/20 | 90 |
|  |  |  |
| Medial Condyle Lesion | 18/20 | 90 |
| Linear hyper-intense T2 signal ^4^ | 8/18 | 44 |
| Cystic changes ^5^ | 14/18 | 78 |
| Disruption of subchondral bone ^6^ | 14/18 | 78 |
| Chondral fissuring ^7^ | 7/18 | 39 |
|  |  |  |
| Lateral Condyle Lesion | 9/20 | 45 |
| Linear hyper-intense T2 signal ^4^ | 4/9 | 44 |
| Cystic changes ^5^ | 6/9 | 67 |
| Disruption of subchondral bone ^6^ | 6/9 | 67 |
| Chondral fissuring ^7^ | 1/9 | 11 |

1. Magnetic resonance imaging (MRI) scans were only available for 20 femurs collected from pigs at 24 weeks of age. Large early-stage epiphyseal OC lesions in subchondral bone and cartilage signals changes were evident in 18 of 20 femurs. See Figure 1 for age-related epiphyseal early-stage OC lesions detected by MRI.

2. MRI characterizations of lesions displaying specific traits of the total lesions assessed in the medial or lateral condyles.

3. The percentage (%) of bones corresponding to each trait is noted.

4. Linear hyper-intense T2 signals are bright spots in MRI images that reveal abnormal tissues associated with inflammation, demyelination, or ischemia due to greater water or protein content.

5. Cystic changes in MRI images are dark, defined regions that represent a fluid-filled mass associated with tissue separations, debris, or extended wall thickness.

6. Disruption of subchondral bone describes subchondral sclerosis beneath cartilage associated with osteoarthritis, trauma, cartilage damage, pain, and lesion progression.

7. Chondral fissuring appears as thin dark lines or cracks in cartilage layers that reflect fluid accumulation extending into subchondral bone tissue.
