# Supplementary material for: Quantitative traits of early-stage osteochondrosis lesions in porcine distal femurs are consistent with skeletal developmental age
Source: JBMR Plus. 2026 May 22;10(7):ziag091. doi: 10.1093/jbmrpl/ziag091 (PMC13318846; doi:10.1093/jbmrpl/ziag091)
Supplement: Final_update_R2_5_15_2026_Supplemental_Tables_ziag091 [file final_update_r2_5_15_2026_supplemental_tables_ziag091.docx]

**Supplemental Tables:**

**Quantitative traits of early-stage osteochondrosis lesions in porcine distal femurs are consistent with skeletal developmental age**

Brittney P. Kokinos MS^1^, Pamela J. Lang MD^2^, Laura A. Amundson PhD^1^, Matthew A. Halanski MD^3^, and Thomas D. Crenshaw PhD^1^

^1^Department of Animal and Dairy Sciences, University of Wisconsin-Madison, Madison, WI 53706 United States;

^2^Department of Orthopedics and Rehabilitation, Shriners Children’s Twin Cities WoodBury, MN 55125 United States;

^3^Department of Child Health, University of Arizona College of Medicine-Phoenix/Phoenix Children’s Hospital United States; Pheonix AZ 85016 United States

**Corresponding author**:

Thomas D. Crenshaw

Email: tdcrensh@wisc.edu

ORCiD ID: 0000-0001-8886-0264

**Authors CRediT:**

BPK: conceptualization, formal analysis, investigation, methodology, resources, software, writing -original draft, writing - review and editing, validation. visualization

PJL: funding acquisition, methodology, writing - review & editing

LAA: funding acquisition, writing - review & editing

MAH: funding acquisition, writing - review & editing

TDC: data curation, funding acquisition, formal analysis, investigation, project administration, supervision, writing - review & editing, resources, validation. visualization

**Disclosures**

**Data availability statement:** All reasonable requests for review of datasets can be made through contacting the corresponding author following publication.

**Funding statement:** Funds to support this project included unrestricted research gift funds for swine nutrition research (Crenshaw). Lang and Halanski contributed research program funds. Amundson is employed by Zinpro Corporation, Eden Prairie, MN that contributed unrestricted research funds that partially supported the study

Supplemental Table S1. Computed tomography (CT) imaging protocol for porcine limbs and live scans^1^.

| Single kV helical CT, GE Medical System, Waukesha, WI | |
| --- | --- |
| Scan Type | Helical |
| Detector Coverage (mm) | 40 |
| Beam Collimation (mm) |  |
| Detector Rows | 64 |
| Detector Configuration | 64 x 0.625 |
| Scan FOV | Large Body |
| Rotation Time (sec) | 0.5 |
| Helical pitch | 0.516 |
| kV | 120 |
| Smart mA or Manual mA | Smart mA |
| Noise Index | 12 |
| Slice Thickness (mm) | 5 |
| Interval (mm) | 2.5 |
| Recon kernel | Standard |
| DFOV | Please adjust according to the object size |
|  |  |
| Second Recon (Helical CT Thin) |  |
| Slice Thickness (mm) | 0.625 |
| Interval (mm) | 0.625 |
| Recon kernel | Bone plus |
| W/L | 3000/300 |

1. Computed tomography (CT) imaging protocol settings were utilized to scan excised limbs collected from porcine specimens at 7, 12, and 24 weeks.

Supplemental Table S2. Magnetic resonance imaging (MRI) protocol for porcine limbs^1^.

Set up = 3 Tesla, 32 channel Torso Coil FULL- lined with chux; Pig prone, feet first

To view in PACS (Radiology Solutions Radiology Station, Change Healthcare, 2019)

***Sequence-Specific Instructions:***

3-plane Localizer – Verify you have the correct side selected (ie, right vs left - start with knee of interest) Offset approximately R or L 110, 48FOV

Coronal PD - Center over knee joint on all 3 planes, cover all bony anatomy, 16FOV, make sure to include OC lesion

Coronal FS Intermediate-Weighted

Sagittal PD - Center over knee joint on all 3 planes, cover all bony anatomy, 16FOV, make sure to include OC lesion

Sagittal FS T2

1. Magnetic resonance imaging (MRI) protocol settings used to scan excised limbs collected from porcine specimens at 7, 12, and 24 weeks.

Supplementary Table S3. Osteochondrosis grading scheme for gross articular surface lesions in growing pigs^1^

| Score | Description |
| --- | --- |
| 0 | Normal |
| 1 | Surface irregularities but no definitive lesion |
| 2 | Articular cartilage collapse/indentation < 5 mm diameter/length |
| 3 | Articular cartilage collapse/indentation > 5 mm and < 10 mm diameter/length |
| 4 | Articular cartilage collapse/indentation > 10 mm diameter/length |
| 5 | Articular cartilage clefting |
| 6 | Articular cartilage clefting with displaced fragment |

1. From Toth et al., *J Anim Sci*. 2016;94(9):3817-3825. doi:10.2527/jas.2015-9950

Supplementary Table S4. Cross-sectional Study. Distribution of subjective visual lesion scores of articular surface lesions in the distal femur at 24 weeks ^1^

| Lesion score ^2^ | MFC ^3^, n | LFC ^3^, n |
| --- | --- | --- |
| 0 | 2/20 | 8/20 |
| 1 | 2/20 | 1/20 |
| 2 | 4/20 | 4/20 |
| 3 | 6/20 | 5/20 |
| 4 | 5/20 | 2/20 |
| 5 | 1/20 | 0/20 |

1. Values represent the number of femurs within each respective condyle location assigned to the respective lesions score of 20 femurs.

2. Subjective scores were assigned a value from 0 to 5 as described in Supplementary Table 3.

3. MFC, medial femoral condyle; LFC, lateral femoral condyle.

Supplemental Table S5. Cross-sectional Study. Additional characterization derived from MRI analysis of epiphyseal early-stage lesions on either the medial or lateral condyle of excised femurs collected at 24 weeks^1^

| MRI Traits | Observations ^1,2^, n | % ^3^ |
| --- | --- | --- |
| Lesions detected by MRI | 18/20 | 90 |
|  |  |  |
| Medial Condyle Lesion | 18/20 | 90 |
| Linear hyper-intense T2 signal ^4^ | 8/18 | 44 |
| Cystic changes ^5^ | 14/18 | 78 |
| Disruption of subchondral bone ^6^ | 14/18 | 78 |
| Chondral fissuring ^7^ | 7/18 | 39 |
|  |  |  |
| Lateral Condyle Lesion | 9/20 | 45 |
| Linear hyper-intense T2 signal ^4^ | 4/9 | 44 |
| Cystic changes ^5^ | 6/9 | 67 |
| Disruption of subchondral bone ^6^ | 6/9 | 67 |
| Chondral fissuring ^7^ | 1/9 | 11 |

1. Magnetic resonance imaging (MRI) scans were only available for 20 femurs collected from pigs at 24 weeks of age. Large early-stage epiphyseal OC lesions in subchondral bone and cartilage signals changes were evident in 18 of 20 femurs. See Figure 1 for age-related epiphyseal early-stage OC lesions detected by MRI.
2. MRI characterizations of lesions displaying specific traits of the total lesions assessed in the medial or lateral condyles.
3. The percentage (%) of bones corresponding to each trait is noted.
4. Linear hyper-intense T2 signals are bright spots in MRI images that reveal abnormal tissues associated with inflammation, demyelination, or ischemia due to greater water or protein content.
5. Cystic changes in MRI images are dark, defined regions that represent a fluid-filled mass associated with tissue separations, debris, or extended wall thickness.
6. Disruption of subchondral bone describes subchondral sclerosis beneath cartilage associated with osteoarthritis, trauma, cartilage damage, pain, and lesion progression.
7. Chondral fissuring appears as thin dark lines or cracks in cartilage layers that reflect fluid accumulation extending into subchondral bone tissue.
